# Surviving cyanide poisoning: A case report highlighting the role of early antidote use

**DOI:** 10.1016/j.toxrep.2025.102127

**Published:** 2025-09-10

**Authors:** Berber C. Hospes, Elise M.A. Slob, Tom K. Brinkman, Sharif M. Pasha, Erik B. Wilms, Hans W.P.M. Overdiek

**Affiliations:** a Department of Clinical Pharmacy, Haaglanden Medical Center, The Hague, the Netherlands; b Department of Clinical Pharmacy, Diakonessenhuis, Utrecht, the Netherlands; c Department of Genetics, University Medical Center Groningen, Groningen, the Netherlands; d Department of Internal Medicine, Erasmus Medical Center, Rotterdam, the Netherlands; e Department of Internal Medicine, Haaglanden Medical Center, The Hague, the Netherlands; f AHZ Farmacie, Laboratory of TDM and Toxicology, The Hague, the Netherlands

**Keywords:** Cyanide, Intoxication, Antidotes, Infrared spectrometry

## Abstract

Survival after high-dose oral cyanide ingestion is rare, and when untreated cases often result in death within an hour. Immediate treatment however, can be lifesaving. We describe a patient who fully recovered after prompt intervention. He arrived at the emergency department unconscious, with red skin and a Glasgow Coma Scale of 3 and was suspected of an intentional intoxication with an unknown white crystalline powder. He rapidly suffered a cardiac arrest. Blood gas analysis showed severe metabolic acidosis, high lactate, and slightly elevated methemoglobin. In suspect of cyanide poisoning, hydroxocobalamin (2 doses of 5 g intravenous) and sodium thiosulfate (12.5 g intravenous) were administered. Thereafter spontaneous circulation returned. The patient was intubated and sedated in the intensive care unit for four days. After extubation, he was transferred to a general ward. A magnetic resonance imaging scan showed no post-anoxic or toxic damage. During his 14-day stay, he fully recovered. The white powder was identified in the pharmaceutical laboratory by infrared spectrometry, confirming the presence of cyanide. Subsequently, the patient admitted to ingesting potassium cyanide. He obtained the potassium cyanide from his workplace, a chemical laboratory.

## Highlights

• Suspect cyanide toxicity in unresponsive, hemodynamically unstable patients with cherry-red skin and severe acidosis.
• Immediate administration of hydroxocobalamin and sodium thiosulfate can be life-saving.
• In order to start immediate treatment, antidotes for cyanide must be in stock at emergency departments
• identification with IR-spectrometry is valuable to confirm the presence of cyanide.

## Graphical Abstract

**Figure:**
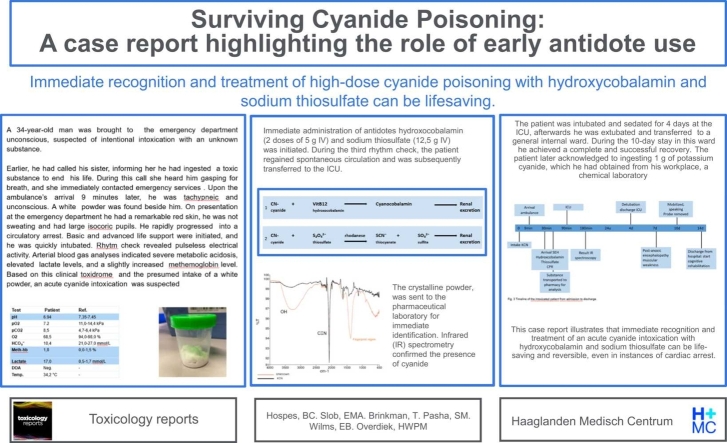

## 1 Introduction

Cyanide is one of the most potent and rapidly acting toxic agents. Naturally occurring sources include almonds, cassava and the kernels of stone fruits such as apricots, cherries, peaches, and plums. Due to its chemical properties, cyanide compounds are widely used in the chemical industry [1]. Acute cyanide intoxication can occur in cases of intentional ingestion or inhalation of hydrogen cyanide in industrial accidents and residential fires.

Cyanide can be fatal within seconds to minutes or hours following systemic exposure after ingestion or inhalation. The lethality of cyanide exposure is influenced by the compound’s chemical form (gaseous, liquid, or solid), route of exposure, duration of contact, and received dose [2], [3]. High-dose oral ingestions, as in suicidal attempts, are often rapidly fatal. Whereas lower-dose exposures (e.g. from almonds or cassava) may allow survival with permanent disabilities, such as the tropical ataxic neuropathy (TAN) and Konzo disease [4]. Early signs and symptoms of acute cyanide poisoning are primarily the body’s reflexive responses trying to counteract tissue hypoxia; increase in blood pressure including hypertension, hyperventilation, shortness of breath and palpitation. Patients often present with dilated pupils lacking light sensitivity. Importantly, several studies by Baud and colleagues have demonstrated that such signs are frequently observed in victims of smoke inhalation, yet often misattributed to carbon monoxide poisoning rather than to concomitant cyanide exposure [5]], [6], [7]. Late symptoms of severe cases shift towards depression of the nervous, respiratory and cardiovascular systems resulting in hypotension, bradycardia, metabolic acidosis, elevated serum lactate, and cardiorespiratory arrest [8]. Toxic effects such as metabolic acidosis start at a plasma lactate concentration of 0.2 mg/L. Neurological symptoms including agitation, seizures, and finally coma, typically developed at concentrations starting at 1 mg/L whereas levels exceeding 2 mg/L are considered potentially fatal [2]. Unmetabolized hydrogen cyanide (HCN) has a bitter almond-like odor that sometimes can be smelled on the breath or from the gastric contents of cyanide-poisoned patients. Rapid recognition and intervention are critical in the management of a cyanide intoxication.

Cyanide toxicity impacts all organs, particularly those highest in oxygen demand like the heart and brain. Poisoning results from the cellular inability to use oxygen while oxygen transport and delivery are still maintained. Cyanide anion (CN-), a diatomic species of similar size to molecular oxygen (O2), binds to the ferric heme moiety of cytochrome c oxidase. This inhibits oxidative phosphorylation and the production of ATP. Disrupted ATP production forces tissues into anaerobic metabolism, leading to excessive lactate accumulation and severe lactic acidosis [2], [3]. The reduced availability of ATP results in cellular dysfunction and cell death.

## 2 Case

A man in his mid-thirties was presented to the emergency department after ingesting an unknown white crystalline powder in an intentional suicide attempt. Earlier he had called his sister, informing her he had ingested a toxic substance to end his life. During this telephone call she heard him gasping for breath. She immediately contacted emergency services. Upon the ambulance’s arrival nine minutes later, he was gasping for breath with an oxygen saturation of 98 %, respiratory rate was 12/min, blood pressure 126/100 mmHg and pulse 80 beats per minute, a Glasgow coma scale of 3 and his body temperature was 35.9 °C

Fifteen minutes later the patient arrived at the emergency department. His pupils were isocoric and unresponsive to light. Light pink foam appeared at the patient's mouth, and he had a remarkable cherry-red skin. There were no signs of diaphoresis, and during handover by the ambulance crew, neither spontaneous respiration nor palpable pulses were observed, prompting the initiation of basic life support (BLS). The ambulance personnel handed over the white coloured powder found at his home.

Arterial blood gas during BLS showed metabolic acidosis with a pH of 6.94, pCO2 of 8.5 mmHg, and bicarbonate of 10.4 mmol/L, resulting in a base excess of −18.8 mmol/L, lactate of 17 mmol/L, methemoglobin of 1.8 % and O2 saturation of 68.5 %.

Because of the sudden deterioration of his clinical symptoms, acute intoxication with a highly toxic systemic asphyxiant, such as cyanide, sodium azide, or sodium nitrite, was suspected. A similar clinical presentation (red skin, metabolic acidosis, asphyxia, respiratory insufficiency, and loss of consciousness) is expected with these substances. Given that higher percentages methaemoglobinaemia would be expected following the ingestion of sodium nitrite, cyanide or sodium azide intoxication was considered the primary diagnosis.

Therefore, empirical treatment was immediately initiated before identification of the powder with the antidote hydroxocobalamin (2 times 5 g intravenous) followed by sodium thiosulphate (12.5 g intravenous). This led to return of spontaneous circulation. Given the suspicion of toxic substance ingestion, endotracheal intubation with a cuffed tube was performed in preparation for gastric lavage, followed by administration of laxatives and activated charcoal. The patient was sedated with midazolam and transferred to the intensive care unit (ICU).

**Fig. 1:**
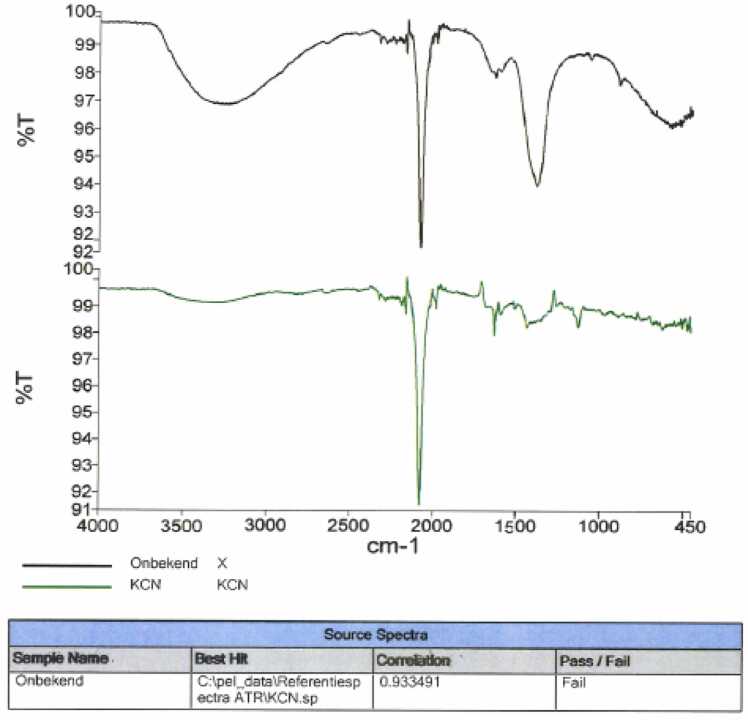
IR spectrum of the unknown substance (the upper black trace)) compared to a potassium cyanide reference sample (KCN) (lower green trace). Infrared spectra were obtained using Fourier transform infrared spectroscopy (FT-IR) on a Spectrum Two spectrometer (PerkinElmer, USA) equipped with an attenuated total reflectance (ATR) accessory. Spectra were recorded in the range 4000–400 cm⁻¹ with a resolution of 4 cm⁻¹ . Samples were analyzed as powders directly applied onto the ATR crystal without further preparation. As a reference, potassium cyanide (KCN) of analytical grade was used.and is presumed to correspond to the Merck “potassium cyanide for analysis” product (purity >97.0 %, CAS 141–50–8), based on an overlapping catalogue number (Merck product no. 1.04967) that was documented at the time. The exact batch documentation, however, is no longer retrievable.

Urine screening for drugs of abuse (benzodiazepines, cannabis, cocaine, opioids, methadone, tricyclic antidepressants, barbiturates, amphetamines) showed negative results. The white powder was sent to the pharmaceutical laboratory for immediate identification in the laboratory using infrared spectrometry (Fig. 1). The powder was identified as (potassium) cyanide.

The patient remained intubated and sedated for 4 days at the ICU. Thereafter, he was extubated and transferred to a medical psychiatric unit for further recovery. His clinical recovery was rapid, allowing for independent intake and safe mobilization. However, cognitive recovery progressed more gradually, though marked improvement was observed during hospitalization. A magnetic resonance imaging scan performed several days after the incident revealed no evidence of post-anoxic damage or toxic metabolic changes. Following a fourteen-day hospital admission, the patient was transferred to a rehabilitation center, where he remained for five weeks to undergo further cognitive rehabilitation. The patient achieved a complete and successful recovery. The patient later acknowledged that he ingested approximately 1 g of potassium cyanide, which he had obtained from his workplace, a chemical laboratory.

## 3 Discussion

Intoxications with cyanide are rare, but effective patient assessment and rapid recognition of symptoms is crucial for rapid establishment of diagnosis. Here, we show a case of successful treatment with hydroxocobalamin after a fatal dose of cyanide ingestion. Although the antidotes used are well established, this case highlights that survival critically depends on the immediacy of recognition and treatment. Moreover, it underscores the value of infrared spectroscopy as a safe and practical tool for confirming cyanide in an unknown powder without the need for manipulations that could release HCN gas, thereby protecting laboratory personnel. In addition, this case also shows that treatment should also be initiated after loss of circulation with a positive effect.

In a review of 1252 reported cases of registered (both acute and accidental) cyanide poisoning, intoxication most commonly resulted from accidental inhalation of cyanide-containing substances or from ingestion of cyanogenic plants [1]. In most patients, the clinical presentation was asymptomatic, and symptoms such as ‘cherry red’ skin or a bitter almond odor were not consistently observed [1], [2], [9], [10]^,^. This may be due to their absence, the smell is only perceptible at 1–5 parts per million, or failure to be recognized. Research shows that 20–40 % of men cannot fully detect the odor because of X-linked recessive variability [2]. Clinicians should keep a high level of suspicion for cyanide toxicity for an unresponsive, hemodynamically unstable patient with significant persistent lactic and metabolic acidosis, with potential access to cyanide salts.

Establishing a rapid diagnosis is challenging due to the interindividual variation in clinical presentation. It is essential to consider both the combination of clinical symptoms and the patient's background. As discussed in our case, the patient worked in a chemical laboratory, having access to toxic substances such as potassium cyanide. Multiple published case reports of work-related ingestion underscore the relevance of the context of the patient, including access through occupations such as jewelry manufacture, the metal industry, or laboratory settings [11]. Which highlights the importance of understanding a patient's background in recognizing acute cyanide poisoning, particularly due to easy access to such toxic substances.

As previously mentioned, the lethality of cyanide exposure depends on the compound’s chemical form (gaseous, liquid, or solid), route of exposure, duration of contact, and the dose received. Absorption following inhalation is rapid and correlates with cyanide concentration in mg/m^3^. Exposure to 120–150 mg/m³ is life-threatening and may result in death within 30–60 min, 150 mg/m³ can be fatal within 30 min, and 200 mg/m³ is likely to cause death within 10 min [12], [13]. The estimated fatal dose of cyanide following oral ingestion is approximately 1.4 mg/kg (calculated as hydrogen cyanide) [12], [13]. Absorption is both rapid and effective, with clinical symptoms generally manifesting between 30 min and 2 h post-exposure. In this case, the patient had ingested 1000 mg. Considering the molecular weight of potassium cyanide (38 g/mol) and that of the cyanide ion (19 g/mol), an ingestion of 1000 mg KCN corresponds to approximately 500 mg of cyanide. Based on a body weight of 95 kg and in comparison to the reported lethal dose (LD₅₀) for hydrogen cyanide of 1.4 mg/kg, the estimated cyanide exposure in this case amounts to approximately 5 mg/kg— well above the estimated lethal threshold. Clinical signs appeared within 30 min, and he survived. This may be explained by the rapid recognition on presentation at the emergency ward in combination with the fact that in some cases of cyanide poisoning a proportion of the ingested substance may remain unabsorbed in the gastrointestinal tract, resulting in a lower effective, systemic dose [14]. The onset of symptoms varies depending on route of exposure. Therefore it is important to identify the specific form of cyanide to which the patient was exposed, as this can significantly influence the clinical course of the intoxication.

Early intervention in cyanide poisoning can be lifesaving. The administration of antidotes such as hydroxocobalamin and sodium thiosulfate is highly effective when administered in a timely manner [15], [16], [17], [18]. Hydroxocobalamin has a favorable tolerability profile [8]. It does not affect blood oxygenation and appears to generally enhance hemodynamic stability in victims of cyanide poisoning. Antidotes for cyanide function by either binding to the cyanide ion directly or indirectly, preventing it from binding to the ferric ion in cytochrome c oxidase, or accelerating its conversion to less toxic substances. High-dose hydroxocobalamin binds cyanide directly to form cyanocobalamin, a stable and nontoxic compound. Sodium nitrite was historically believed to act mainly through the induction of methemoglobinemia, allowing ferric hemoglobin to bind cyanide. More recent evidence, however, demonstrates that its primary antidotal activity is mediated via nitric oxide (NO) release, which can reverse cyanide inhibition of cytochrome c oxidase. Formation of methemoglobin may still occur, but this is considered a secondary and clinically less relevant mechanism [19]. Because of its direct binding mechanism and favorable safety profile, hydroxocobalamin is regarded as first-line therapy in severe cyanide intoxication. Sodium thiosulfate acts through a distinct pathway, serving as a sulfur donor for rhodanese and related sulfurtransferases, thereby accelerating the conversion of cyanide into the less toxic, renally excreted thiocyanate [20]. Although this reaction proceeds relatively slowly, its detoxification capacity is high, and sodium thiosulfate can be administered as monotherapy in mild cases of cyanide intoxication or as an adjunct to other antidotes in severe cases.

Determining cyanide in blood remains challenging due to the limited availability of an analytical method. We used infrared spectrometry to analyze the white powder. The absorption peak in the frequency range of 2100–2260 cm−1 indicated a C≡N (carbon to nitrogen triple bond), confirming the presence of cyanide before the patient had fully recovered (Fig. 1). The region of 3000–3500 cm⁻¹ (the OH stretch) shows a broad peak that is present in both the reference substance (KCN) and the unknown powder sample [21], [22]. Since KCN is hygroscopic and naturally absorbs water from the atmosphere, the presence of this peak is expected and normal, regardless of storage conditions. The IR spectrum of the white powder is consistent with cyanide, as evidenced by the presence of a sharp C≡N stretching band around 2100 cm⁻¹ . To minimize risks associated with inadvertent exposure to HCN gas, all samples should be handled in sealed containers during analysis and care should be taken to avoid contact with aqueous solutions during sampling or analysis.

The analysis confirmed the diagnosis of a cyanide intoxication. In practice, due to the toxicity of cyanide and the rapid onset of symptoms, it is not feasible to wait for these results before making a diagnosis. In this case, treatment was initiated immediately upon presentation. There are few documented cases of patients who have survived an intentional intoxication with this amount of cyanide. This case report demonstrates that rapid intervention can lead to full recovery, even in cases of exposure to a potential fatal dose. Additionally, the unknown substance was rapidly identified using infrared spectroscopy, a helpful diagnostic approach not previously reported in the literature in case of a cyanide intoxication. This technique played a crucial role in confirming the diagnosis.

One limitation of this report is the absence of confirmation of serum cyanide level measurements. However, this test is not typically used as a routine diagnostic tool in cyanide poisoning due to limited availability of a validated method and its minimal impact on immediate clinical decision-making.

## 4 Conclusion

This case report shows that immediate recognition and treatment of acute cyanide poisoning with hydroxycobalamin and sodium thiosulfate can be lifesaving. Clinicians should keep a high level of suspicion for cyanide toxicity for an unresponsive, hemodynamically unstable patient with cherry-red skin, cardiorespiratory arrest, significant persistent lactic and metabolic acidosis, with potential access to cyanide salts. Given the rapid onset of toxicity, immediate administration of antidotes and symptom management is crucial. If the ingested substance is available, infrared spectroscopy can confirm the diagnosis. However, given the urgency, start treatment promptly. Emergency departments should be equipped with antidote kits and protocols for effective cyanide toxicity management to improve outcomes.

## CRediT authorship contribution statement

**Hospes, Berber C.:** Writing – original draft, Writing – review & editing, , Investigation, Conceptualization. **Slob, Elise M.A.:** Writing – review & editing, Conceptualization. **Brinkman, Tom:** Writing – review & editing, Investigation, Conceptualization. **Pasha, Sharif M.:** Writing – review & editing, Conceptualization. **Wilms, Erik B.:** Writing – review & editing, Conceptualization. **Overdiek, Hans W.P.M.:** Writing– review & editing, Conceptualization.

## Declaration of Competing Interest

The authors declare that they have no known competing financial interests or personal relationships that could have appeared to influence the work reported in this paper.

## Data Availability

Data will be made available on request.
